# The *ABCG2* rs223114*2* polymorphism and the risk of nephrolithiasis: A case–control study from the Taiwan biobank

**DOI:** 10.3389/fendo.2023.1074012

**Published:** 2023-03-10

**Authors:** Ching-Tsai Lin, I-Chieh Chen, Yen-Ju Chen, Ying-Cheng Lin, Jui-Chun Chang, Tsai-Jung Wang, Wen-Nan Huang, Yi-Hsing Chen, Yi-Huei Chen, Ching-Heng Lin, Yi-Ming Chen

**Affiliations:** ^1^ Division of Allergy, Immunology and Rheumatology, Department of Internal Medicine, Taichung Veterans General Hospital, Taichung, Taiwan; ^2^ School of Medicine, National Yang Ming Chiao Tung University, Taipei, Taiwan; ^3^ Department of Medical Research, Taichung Veterans General Hospital, Taichung, Taiwan; ^4^ Division of Gastroenterology and Hepatology, Department of Internal Medicine, Taichung Veterans General Hospital, Taichung, Taiwan; ^5^ Department of Obstetrics and Genecology and Women’s Helath, Taichung Veterans General Hospital, Taichung, Taiwan; ^6^ Division of Nephrology, Department of Internal Medicine, Taichung Veterans General Hospital, Taichung, Taiwan; ^7^ Department of Critical Care Medicine, Taichung Veterans General Hospital, Taichung, Taiwan; ^8^ Department of Post-Baccalaureate Medicine, College of Medicine, National Chung Hsing University, Taichung, Taiwan; ^9^ Department of Public Health, College of Medicine, Fu Jen Catholic University, New Taipei City, Taiwan; ^10^ Department of Health Care Management, National Taipei University of Nursing and Health Sciences, Taipei City, Taiwan; ^11^ Department of Industrial Engineering and Enterprise Information, Tunghai University, Taichung, Taiwan; ^12^ Institute of Public Health and Community Medicine Research Center, National Yang Ming Chiao Tung University, Taipei City, Taiwan; ^13^ Department of Internal Medicine, China Medical University Hospital, Taichung, Taiwan; ^14^ Institute of Biomedical Science and Rong Hsing Research Center for Translational Medicine & Program in Translational Medicine, National Chung Hsing University, Taichung, Taiwan; ^15^ Precision Medicine Research Center, College of Medicine, National Chung Hsing University, Taichung, Taiwan

**Keywords:** nephrolithiasis, *ABCG2* rs2231142, predictors, single-nucleotide polymorphism, precision healthcare

## Abstract

**Background:**

Hyperuricemia and gout are risk factors of nephrolithiasis. However, it is unclear whether the *ABCG2* gene contributes to the development of nephrolithiasis. We aimed to investigate the interaction between the *ABCG2* rs2231142 variant and incident nephrolithiasis in the Taiwanese population.

**Methods:**

A total of 120,267 adults aged 30–70 years were enrolled from the Taiwan Biobank data-base in this retrospective case–control study and genotyped for rs2231142. The primary outcome was the prevalence of self-reported nephrolithiasis. The odds ratio (OR) of incident nephrolithiasis was analyzed by multivariable logistic regression models with adjustment for multifactorial confounding factors. Associations of the *ABCG2* rs2231142 variant with serum uric acid levels, and the incident nephrolithiasis were explored.

**Results:**

The frequency of rs2231142 T allele was 53%, and 8,410 participants had nephrolithiasis. The multivariable-adjusted OR (95% confidence interval) of nephrolithiasis was 1.18 (1.09–1.28) and 1.12 (1.06–1.18) for TT and GT genotypes, respectively, compared with the GG genotype (*p*<0.001), specifically in the male population with hyperuricemia. Higher age, male sex, hyperlipidemia, hypertension, diabetes mellitus, hyperuricemia, smoking and overweight were independent risk factors for nephrolithiasis. In contrast, regular physical exercise is a protective factor against nephrolithiasis.

**Conclusions:**

*ABCG2* genetic variation is a significant risk of nephrolithiasis, independent of serum uric acid levels. For rs2231142 T allele carriers, our result provides evidence for precision healthcare to tackle hyperuricemia, comorbidities, smoking, and overweight, and recommend regular physical exercise for the prevention of nephrolithiasis.

## 1 Introduction

Nephrolithiasis (kidney stones) is a common disease that clinically present as acute colicky pain and often recurs in association with morbidities. Worldwide, the prevalence of nephrolithiasis ranges from 1% to 19.1% in Asia, 5% to 10% in Europe, 4% in South Africa, 7% to 13% in North America, and as 20% to 25% in the Middle East (1–3). In the United States, the 7.4% absolute increase in the prevalence of self-reported nephrolithiasis over four decades – from 3.2% in 1980 to 10.6% in 2018 is noteworthy (4, 5). In China, the prevalence of nephrolithiasis almost doubled from 5.95% in 1991–2000 to 10.63% in 2011 (6). The 5–15% increase in the prevalence of nephrolithiasis in developed and developing countries during the past few decades has increased of health-care cost by approximately 50%, which includes both the direct treatment costs and the indirect costs associated with loss of work productivity (3, 7). Incident and recurrent nephrolithiasis confers a high risk of comorbidity that results in acute and chronic renal failure (8). Despite the availability of various therapeutic approaches, up to 50% and 75% of patients with nephrolithiasis develop recurrence within 5–10 years and 20 years, respectively (9).

Significant predisposing factors of nephrolithiasis can be classified into five categories: lifestyle, genetics, diet, environment, and systemic comorbidities (3, 10). The contribution of aforementioned risk factors to nephrolithiasis varies in different populations. Moreover, biochemical abnormalities in the urinary composition have been associated with the risk of nephrolithiasis (10). Nephrolithiasis characteristically includes four phenotypes – calcium, urate, struvite (magnesium ammonium phosphate), and cystine stones (10). Approximately 80% of nephrolithiasis, either homogenously or heterogeneously is constituted by the commonest phenotypes of calcium oxalate (CaOx) or calcium phosphate (11). Comprising less than 10% of cases, urate stones constitute the third most common type of nephrolithiasis and are attributed to predisposing factors such as persistently low urinary pH level, hypovolemia (decreased urinary volume), and hyperuricosuria (12). Moreover, the dissolved urate in the native urine can salt out CaOx in those with hyperuricosuria and hypercalciuria (13). Allopurinol may effectively prevent the hyperuricosuric and hypercalciuric patients from recurrence of calcium nephrolithiasis (13, 14). However, it is unclear whether hyperuricemia contributes to the development of calcium nephrolithiasis.

In genome-wide association studies (GWAS), the strong association of the *ABCG2* rs2231142 variant with gout that is mediated by both decreased intestinal urate excretion and renal overload hyperuricemia was confirmed in the Asian population (15, 16). Additionally, obesity, diet, lifestyle, genetics and underlying comorbidities are important risks factors that predispose to hyperuricemia and contribute to the pathogenesis of gout (17). The risk of hyperuricemia was markedly increased in the Taiwanese population through the interaction of the rs2231142 variant with obesity (18). Systematic reviews of the predisposition to kidney stones revealed a significant link between obesity, associated disease, and hyperuricosuria (3, 10, 11). However, it is unclear whether the rs2231142 variant plays a crucial role in the phathogenesis of kidney stone, and the interaction between genetic factors and other risk factors of nephrolithiasis remains elusive.

In this study, we aimed to explore the association between *ABCG2* rs2231142 variant and the risk of nephrolithiasis in a community-based Taiwanese population. The primary objective of the study was to determine the prevalence of self-reported nephrolithiasis in Taiwan and to identify the associations between the *ABCG2* rs2231142 variant and incident nephrolithiasis with hyperuricemia.

## 2 Materials and methods

### 2.1 Study design, participants and ethics statement

This retrospective case–control study was undertaken using the data collected in the Taiwan Biobank (TWB) database between September 2014 and May 2021 and included 120,267 adult Taiwanese Han Chinese participants aged 30–70 years. The TWB is a prospective population-based research project to recruit volunteers from the general population. Participants with a history of cancer were excluded from enrollment. To study the complex interaction between genomics and comorbidities of public importance, subjects with chronic diseases were not excluded. All participants from 29 recruitment medical centers in Taiwan provided written informed consent before the sample collection and the subsequent analysis. The TWB datasets used and/or analyzed in this study comprised specimens and information that were collected using a completely standardized procedure to fit researchers’ needs in different fields (19, 20). For this study, we obtained genotyping information, demographics (i.e., sex and age), medical history, lifestyle modalities (i.e., alcohol consumption, smoking and physical activity), physical examination (i.e., body mass index [BMI; kg/m^2^], and blood pressure [BP; mmHg]), and biochemical reports (i.e., serum uric acid [UA], creatinine, fasting glucose levels, total cholesterol [TC], triglyceride [TG], high-density lipoprotein cholesterol [HDL-C], low-density lipoprotein cholesterol [LDL-C] in mg/dL etc.) from the TWB database in conformance with the Declaration of Helsinki and with ethical approval by the Institutional Review Board of Taichung Veterans General Hospital, Taichung, Taiwan (IRB No. CE16270B-2).

### 2.2 Genotyping and quality controls

Blood samples for DNA analysis were collected from TWB participants and the genotype was determined by using the Axiom Genome-Wide TWB array (Affymetrix, Santa Clara, CA, USA) at the National Center for Genome Medicine in Academia Sinica, Taiwan (19, 20). TWB implemented Affymetrix Power Tools (APT) as a standard quality-control procedure to select specific single-nucleotide polymorphisms (SNPs) that are suitable for analyzing the genetic traits of the Taiwanese Han Chinese populations. SNPs on the X and Y chromosomes, as well as those on mitochondrial DNA, were included for data release (19). In total, 653,291 SNPs were included in the Affymetrix TWB 2.0 SNP chip. Details about the TWB are available from the official website (https://taiwanview.twbiobank.org.tw/index). PLINK was used for analyzing and working with Affymetrix microarray data, as well as for controlling the quality of the procedure with the Hardy–Weinberg equilibrium test (21).

### 2.3 Data collection and outcome identification

Data on medical and family history, personal history, and history of systemic comorbidity were collected in a self-reported questionnaire that was completed during a face-to–face interview. conducted by a trained interviewer. The primary outcome was the prevalence of self-reported nephrolithiasis that was ascertained from the history/-treatment of nephrolithiasis (4–6). Accordingly, we enrolled 120,267 individuals (44,151 men and 76,116 women) for whom information of genotyping and nephrolithiasis were available. Among them, 5,086 men and 3,324 women with self-reported nephrolithiasis identified from TWB database were included in the case group. The remaining 111,857 (39,065 male and 72,792 female) participants without a history or family history of nephrolithiasis were included in the control group.

The relevant biochemical and lifestyle data of the participants were extracted from the TWB database, and the following covariates were evaluated: sex, age, BMI, alcohol consumption, smoking, physical activity, serum UA levels and systemic comorbidity.

BMI ≥24 kg/m^2^ was defined as overweight for the East Asian population. Habitual alcohol consumption was defined as the intake of more than 150 mL alcohol per week for at least 6 months and smoking was defined as daily use of tobacco continuously for at least 6 months. The extent of physical activity was dichotomized as non-regular and regular exercise (>30 minutes of exercise at least three times a week). Systemic comorbidity included hyperlipidemia, which was defined based on administration of lipid-lowering therapy or a physician’s diagnosis is based on objective parameters (TC ≥200 mg/dL, LDL-C ≥ 130 mg/dL, TG ≥ 150 mg/dL, or HDL-C < 40 or <50 mg/dL in men or women respectively), hypertension (systolic and/or diastolic BP ≥ 140/90 mmHg), diabetes mellitus (receiving hypoglycemic agents, HbA1c ≥ 6.5%, random plasma glucose ≥200 mg/dL, fasting plasma glucose ≥ 126mg/dL, and/or diagnosed by physicians), and hyperuricemia (serum UA level >7.0 mg/dL measured by the uricase method using an Architect i2000SR Analyzer, Abbott Diagnostics, Abbott Park, Chicago, IL, United States).

### 2.4 Statistical analysis

Quantitative variables are expressed as the mean ± standard deviation (SD). Mann-Whitney U test for continuous variables and Chi-square test for categorical variables were conducted to compare variables between nephrolithiasis cases and non-nephrolithiasis controls. In the sex-stratified analysis, statistical differences between the cases and controls with the rs2231142 genotypes and relationships between categorical variables were analyzed by the chi-square test. The associations between the *ABCG2* rs2231142 variant and incident nephrolithiasis with hyperuricemia was analyzed using a multivariable-adjusted logistic regression model for potential confounding factors. Odds ratios (OR) and 95% confidence intervals (CI) were calculated. All statistical analysis was performed in SAS version 9.4 (SAS Institute Inc. Cary NC). Significance was set at *p*<0.05 and *p*<0.005 when appropriate. *Post-hoc* analysis with Bonferroni correction was utilized to reduce the chance of false-positive results in multiple pairwise tests.

## 3 Results

### 3.1 Demographics at baseline and incident nephrolithiasis

Among the 120,267 participants aged 30–70 years, including 44,151 men and 76,116 women, 8,410 participants were identified with nephrolithiasis (prevalence: 11.52% in men and 4.37% in women). The characteristics of the participants are shown in **Table 1**. The mean age of the cases was significantly higher than that of the controls (53.6 ± 9.8 vs. 49.7 ± 11.0, *p*<0.001), and men had significantly higher risk of nephrolithiasis than women (60.5% vs. 39.5%, *p*<0.001). Compared with the GG genotype, the *ABCG2* rs2231142 TT (11.1% vs. 9.9% *p*<0.001) and GT (45.0% vs.42.9%, *p*<0.001) genotypes were more frequently observed in the cases. The prevalence of hyperlipidemia (14.8% vs. 7.0%, *p*<0.001), hypertension (26.3% vs. 11.4%, *p*<0.001), diabetes mellitus (10.0% vs. 5.0%, *p*<0.001), and hyperuricemia (23.0% vs. 12.7%, *p*<0.001) were significantly higher in the nephrolithiasis group than in the controls. Moreover, lifestyle factors including smoking (41.3% vs. 26.4%, *p*<0.001), alcohol consumption (8.7% vs. 5.8%, *p*<0.001), regular physical exercise (44.8% vs. 40.2%, *p*<0.001) and overweight (61.5% vs. 46.8%, *p*<0.001) were significantly higher in the cases compared with that in the controls.

**Table 1:** Stratification of participant characteristics based on the presence or absence of nephrolithiasis.

Variables	Without nephrolithiasis (n=111,857)		With nephrolithiasis (n=8,410)		*p*-value
	n	(%)	n	(%)	
Age, years^a^	49.7 ± 11.0		53.6 ± 9.8		<0.001
Sex^b^					<0.001
female	72,792	(65.1)	3,324	(39.5)	
male	39,065	(34.9)	5,086	(60.5)	
Overweight (BMI≧24 kg/m^2^)^b^					<0.001
No	59,511	(53.2)	3,233	(38.5)	
Yes	52,276	(46.8)	5,166	(61.5)	
*ABCG2* rs2231142^b^					<0.001^*^
GG	45,642	(47.3)	3,206	(43.9)	
GT	41,387	(42.9)	3,291	(45.0)	
TT	9,536	(9.9)	812	(11.1)	
Hyperlipidemia^b^					<0.001
No	104,044	(93.0)	7,162	(85.2)	
Yes	7,813	(7.0)	1,248	(14.8)	
Hypertension^b^					<0.001
No	99,130	(88.6)	6,202	(73.7)	
Yes	12,727	(11.4)	2,208	(26.3)	
Diabetes mellitus^b^					<0.001
No	106,270	(95.0)	7,568	(90.0)	
Yes	5,587	(5.0)	842	(10.0)	
Uric acid (mg/dL)^b^					<0.001^*^
<5	46,831	(41.9)	2,244	(26.7)	
5-7	50,742	(45.4)	4,227	(50.3)	
>7	14,208	(12.7)	1,933	(23.0)	
Smoking^b^					<0.001
No	82,243	(73.6)	4,931	(58.7)	
Yes	29,570	(26.4)	3,476	(41.3)	
Alcohol consumption^b^					<0.001
No	105,309	(94.2)	7,669	(91.3)	
Yes	6,453	(5.8)	731	(8.7)	
Regular physical exercise^b^					<0.001
No	66,907	(59.8)	4,636	(55.2)	
Yes	44,895	(40.2)	3,768	(44.8)	

a Continuous variables were expressed as mean ± standard deviation (SD) and analyzed using the Mann-Whitney U test.

b Categorical variables were expressed as numbers (percent) and analyzed using the Chi-square test.

* p-value after Bonferroni correction. BMI: body mass index

### 3.2 Sex-related differences in nephrolithiasis across the rs2231142 genotype categories

**Figure 1:**
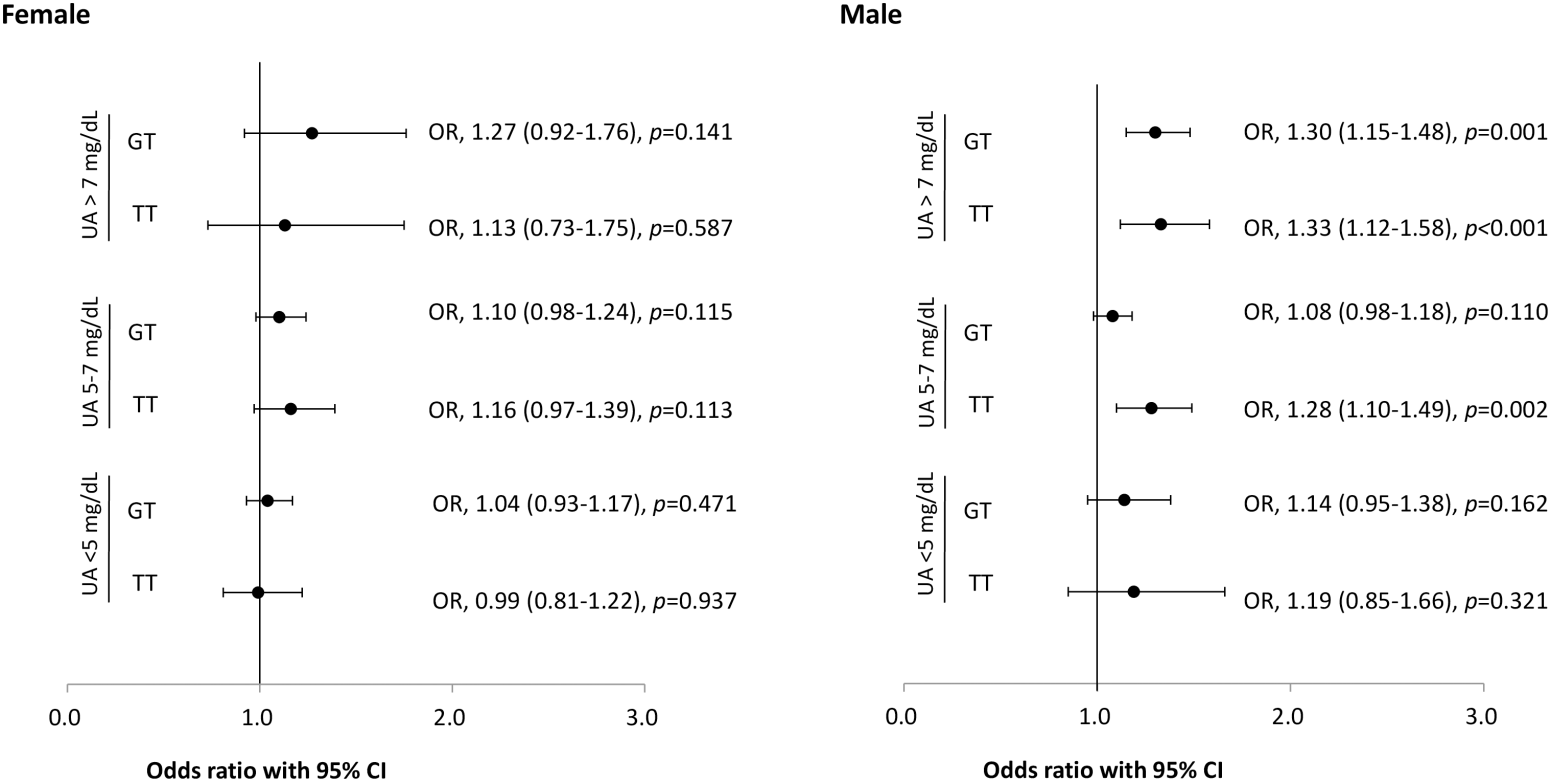
Risk of nephrolithiasis and *ABCG2* rs2231142 genotypes stratified by sex and serum uric acid levels. Error bars represent the 95% confidence intervals (CI) of the odds ratios (OR). UA: uric acid.

The sex-stratified frequency of the *ABCG2* rs2231142 genotype and association with nephrolithiasis is shown in **Table 2**. Significantly more TT (10.5% vs. 9.9%) and GT (44.8% vs. 43.0%) genotypes were found in the female population with nephrolithiasis compared with female controls (*p*<0.05). A similar distribution of the *ABCG2* rs2231142 variants in the male population with nephrolithiasis was observed, with TT and GT genotypes occurring more frequently in participants with nephrolithiasis (*p*<0.001). The serum UA- and sex-stratified association between the rs2231142 genotype and nephrolithiasis are shown in **Figure 1**. The OR of incident nephrolithiasis in the male study cohort with UA >7mg/dL was 1.30 (95% CI: 1.15–1.48, *p*=0.001) and 1.33 (95% CI: 1.12–1.58, *p<*0.001) for GT and TT carries, respectively, and with UA 5–7mg/dL was 1.28 for TT carriers (95% CI: 1.10–1.49, *p=*0.002).

**Table 2:** Sex-stratified risk of nephrolithiasis with the *ABCG2* rs2231142 genotypes.

Variables	Female					Male				
	Without nephrolithiasis (n=62,971)		With nephrolithiasis (n=2,903)		*p*-value	Without nephrolithiasis (n=33,594)		With nephrolithiasis (n=4,406)		*p*-value
	n	(%)	n	(%)		n	(%)	n	(%)	
*ABCG2* rs2231142					0.042					<0.001
GG	29,657	(47.1)	1,298	(44.7)		15,985	(47.6)	1,908	(43.3)	
GT	27,060	(43.0)	1,301	(44.8)		14,327	(42.6)	1,990	(45.2)	
TT	6,254	(9.9)	304	(10.5)		3,282	(9.8)	508	(11.5)	

Data are expressed as number (percentage).

The relationship between categorical variables was ascertained by the chi-square test.

### 3.3 Association of demographics, comorbidities, the *ABCG2* rs2231142 variant, and lifestyle factors, with the risk of nephrolithiasis

To evaluate the association of demographics, comorbidities, rs2231142 variant, lifestyle factors with nephrolithiasis, we created a multivariable-adjusted logistic regression model (**Table 3**). In comparison with the GG genotype, the multivariable-adjusted OR (95% CI) of the GT and TT genotypes for the risk of nephrolithiasis was 1.12 (1.06–1.18) and 1.18 (1.09–1.28), respectively (*p*<0.001 for both). Moreover, the OR (95% CI) of incident nephrolithiasis was 1.03 (1.02–1.03), 2.21 (2.08–2.36), 1.40 (1.29–1.51), 1.67 (1.57–1.78), 1.16 (1.06–1.26), 1.25 (1.16–1.36), 1.15 (1.09–1.22), and 1.22 (1.15–1.28) for higher age, male sex, hyperlipidemia, hypertension, diabetes mellitus, hyperuricemia, smoking, and overweight, respectively (*p*<0.001 for all, except *p*=0.002 for diabetes mellitus). Interestingly, regular physical exercise is a protective factor against nephrolithiasis (OR=0.95, 95% CI: 0.9–1.0, *p*=0.048).

**Table 3:** Association of demographics, comorbidities, *ABCG2* rs2231142 variants, and lifestyle factors with the risk of nephrolithiasis.

Variables	OR	95% CI	*p*-value
Age, years	1.03	(1.02-1.03)	<0.001
Sex			
female	1.00	─	─
male	2.21	(2.08-2.36)	<0.001
Overweight (BMI ≥24 kg/m^2^)			
No	1.00	─	─
Yes	1.22	(1.15-1.28)	<0.001
*ABCG2*rs2231142			
GG	1.00	─	─
GT	1.12	(1.06-1.18)	<0.001
TT	1.18	(1.09-1.28)	<0.001
Hyperlipidemia			
No	1.00	─	─
Yes	1.40	(1.29-1.51)	<0.001
Hypertension			
No	1.00	─	─
Yes	1.67	(1.57-1.78)	<0.001
Diabetes mellitus			
No	1.00	─	─
Yes	1.16	(1.06-1.26)	0.002
Uric acid (mg/dL)			
<5	1.00	─	─
5-7	1.07	(1.01-1.14)	<0.026
>7	1.25	(1.16-1.36)	<0.001
Smoking			
No	1.00	─	─
Yes	1.15	(1.09-1.22)	<0.001
Alcohol consumption			
No	1.00	─	─
Yes	0.92	(0.84-1.01)	0.083
Regular physical exercise			
No	1.00	─	─
Yes	0.95	(0.90-1.00)	0.048

Results obtained from multivariable logistic regression analyses.

Odds ratio (OR) adjusted for all variables in the table; BMI, body mass index; CI, confidence intervals.

-, not applicable.

## 4 Discussion

In this study, we confirmed the importance of the *ABCG2* rs2231142 variant with which the risk of nephrolithiasis is significantly associated. The risk of incident nephrolithiasis increased with hyperlipidemia, hypertension, diabetes mellitus, and hyperuricemia as well as with lifestyle factors (overweight and smoking) rather than active alcohol consumption. Regular physical exercise conferred a protective effect against nephrolithiasis.

The lifetime prevalence of nephrolithiasis increases with age. The highest prevalence of nephrolithiasis in the general population was observed in those over 60 years in the United States and in Asia individuals aged 30–60 years (1, 5). The prevalence of nephrolithiasis showed a sex difference and was more common among men than in women, with a declined ratio to 1.26 in the United States and 1.3–5.0 in Asia (1, 5). As the rates of overweight and metabolic syndrome increased significantly, they promoted an increase of 2.9% nephrolithiasis among US women from 2007 through 2018 (5, 22). In the Taiwanese population, the prevalence of nephrolithiasis that was ascertained using the National Health Insurance Research Database in 2010 was 7.38% in the entire cohort, with a male predominance (9.01% vs. 5.79% [in women]) (23). In the present study, the mean age at onset of nephrolithiasis was 53.6 ± 9.8 years, and men were more likely to have nephrolithiasis than women (OR=2.21).

The *ABCG2* rs2231142 genotype has a strong association with hyperuricemia and gout through the mechanisms of both decreased intestinal urate excretion and renal overload in the Asian population (15, 16). The history of gout was evidently linked to a higher prevalence of nephrolithiasis (5). The *ABCG2* gene contributes to inflammation in gouty arthritis that is mediated *via* the release of Interleukin 8 (IL-8) following MSU crystals-stimulation in an endothelial cell model (24). Furthermore, the *ABCG2* gene played a crucial role in the aberrant generation of pro-inflammatory cytokines such as interleukin-1 beta (IL-1β), tumor necrosis factor-alpha (TNF-α) and IL-8 in gout. The oxidative stress triggered by reactive oxygen species (ROS) and pro-inflammatory cytokines results in gouty arthropathy, and also leads to renal damage in the kidney (25). Moreover, the rs2231142 T-allele was associated with early-onset gout and tophaceous disease in Western Polynesian individuals with gout (16, 26). A 10-year observational study showed significantly higher tophaceous gout in the early-onset group (27). The expression of pro-inflammatory cytokines and receptor activator of nuclear factor κB ligand (RANKL) in T-cell induced by the MSU crystals of chronic gouty arthritis leads to osteoclastogenesis (28). These mechanisms occur in gout with a 1.2-fold increased risk of osteoporosis (29) which may play an independent risk factor for development of nephrolithiasis (30). Our study is the first to reveal that the risk of nephrolithiasis was markedly increased in participants with the *ABCG2* rs2231142 T genotypes. Our finding concurs with the results in previous studies and suggests that the association between the *ABCG2* genetic variants may contribute to the formation of nephrolithiasis through inflammation elicited by hyperuricemia and gout.

Dyslipidemia is an independent risk factor, not only for recurrent multi-stone nephrolithiasis but also for abnormalities in urinary constituents (31, 32). In addition, a study from NorthShore University Health System showed that incident nephrolithiasis significantly increased with higher TG levels and could be partly prevented by statin use (33). In the present study, we discovered a significant association between hyperlipidemia and incident nephrolithiasis. Hypertension may contribute to the incidence of nephrolithiasis through increased urinary calcium excretion (34). In addition, the risk of hypertension increased by 58% after the first symptomatic kidney stone event (35). Although the biological mechanism underlying the association between hypertension and nephrolithiasis is unknown, there appears to be a bidirectional relationship (34, 35). Consistent with the results of a previous study, this study demonstrated that hypertension is significantly associated with incident nephrolithiasis (36). Furthermore, insulin resistance may lead to lower urinary pH values in the renal proximal tubule and hyperuricosuria, which is linked to nephrolithiasis (37). The urate stones were more frequently observed in patients with diabetes mellitus than in patients without diabetes (38). In this study, we discovered a significant association between type 2 diabetes and nephrolithiasis, and found that hyperuricemia is an independent risk factor for incident nephrolithiasis. Asymptomatic hyperuricemia was an independent risk factor for nephrolithiasis in a large cohort study (39). Hyperuricosuria, low urinary pH level, and low urinary volume (dehydration) were crucial drivers that promoted nephrolithiasis (3, 11). A possible mechanism underlying the aforementioned relationship is that the dissolved sodium urate could salt out CaOx from native urine (13). Taken together, our data supported an independent role of genetic factors in nephrolithiasis after controlling for potential contributing comorbidities. Further investigations are needed to elucidate the pathogenesis of *ABCG2* in nephrolithiasis.

Smoking is associated with oxidative stress and leads to renal damage and chronic kidney disease (40). Moreover, crystallization modulators, such as osteopontin, bikunin and α-microglobulin, could be generated by smoking and thus contribute to lithogenesis in the kidneys (41). We discovered an association between incident nephrolithiasis and cigarette smoking, rather than with alcohol consumption. This result was supported by another study which demonstrated that current smoking, but not active alcohol use, was an attributable risk for incident calcium urolithiasis (42).

It is well-known that BMI is associated with biomarker of systemic inflammation of the metabolic syndrome (43). A previous study demonstrated that the prevalence of overweight and obesity was significantly higher in patients with urolithiasis (44). In addition, overweight and obesity are liked to insulin resistance; lower urinary pH level; and excretion of more urate, sodium, calcium, oxalate and phosphate in urine (44, 45). The excess dietary intake of lithogenic substances and a lithogenic urinary profile may predispose obese patients to nephrolithiasis (44). Therefore, it would be beneficial to implement dietary modifications and control comorbidities to reduce the risk of nephrolithiasis in obese patients.

We discovered a protective association between incident nephrolithiasis and regular physical exercise. During physical exercise, body fluid is lost and the sensation of thirst could induce increased water intake. Moreover, sodium is lost through diaphoresis in exercise and resulted in an approximately 50% reduction in the excretion of urinary sodium and decreased the urinary output (46). Collectively, these mechanisms may lead to an expansion of 20–25% of the circulating blood volume and reduce sympathetic tone and cardiovascular diseases after exercise (46). Furthermore, resistance exercise confers a benefit by decreasing urinary calcium excretion (47). Our result supports and advocates that regular physical activity could reduce incident nephrolithiasis (48).

The *ABCG2* rs2231142 variants not only lead from hyperuricemia to gouty arthropathy, but also increase the risk of incident nephrolithiasis. Thus, the screening of *ABCG2* rs2231142 variants is as important as the evaluation of systemic comorbidities and lifestyle factors for patients with nephrolithiasis and hyperuricemia. We infer that the first choice in urate-lowering therapy for those with nephrolithiasis, the *ABCG2* rs2231142 T allele, and gout should be xanthine oxidase inhibitors rather than an uricosuric agent.

There are some limitations of this study that to be mentioned. First, this study was a cross-sectional design from TWB database. However, all of the risk factors for nephrolithiasis have been prospectively collected. We believe that *ABCG2* rs2231142 T-allele might contribute to the formation of nephrolithiasis through inflammation elicited by hyperuricemia and gout. Second, we did not include all lifestyle risk factors such as detailed dietary information and that could affect the risk of nephrolithiasis. Nevertheless, our data support weight control and smoking cessation to mitigate the risk of nephrolithiasis. Third, the occurrence of nephrolithiasis was collected by self-reported history without confirmation of medical review and the urine chemistry and stone composition were unavailable. Therefore, we cannot exclude the possibility of information bias. Moreover, the impact of concomitant urate-lowering agents was not taken into consideration. However, our result provides robust evidence that *ABCG2* rs2231142 genetic variations are associated with both hyperuricemia and incident nephrolithiasis.

## 5 Conclusion

This retrospective case-control study using data from TWB revealed that besides higher age, males sex, smoking, overweight, comorbidities and hyperuricemia, the *ABCG2* rs2231142 genotypes was independently associated with nephrolithiasis. To implement precision healthcare, a test of *ABCG2* rs2231142 polymorphism could offer additional guidance for lifestyle modification in the prophylactic management of patients with multiple systemic comorbidities and hyperuricemia.

## Data availability statement

The original contributions presented in the study are included in the article/**Supplementary Material**. Further inquiries can be directed to the corresponding author.

## Ethics statement

This research project was approved by the ethics committee of Taichung Veterans General Hospital Institutional Review Board (IRB No. CE16270B-2). The patients/participants provided their written informed consent to participate in this study.

## Author contributions

C-TL and Y-MC conceptualized the study. C-TL, Y-HuC, I-CC, C-HL, and Y-MC were responsible for data curation. Y-HuC, and I-CC were responsible for formal analysis. Y-MC and C-HL were responsible for funding acquisition. Y-JC, Y-CL, J-CC, T-JW, W-NH, and Y-HsC were responsible for investigation. C-TL, W-NH, Y-HsC, C-HL, and Y-MC were responsible for methodology. Y-JC, Y-CL, J-CC, and T-JW, C-HL, and Y-MC were responsible for the resources. W-NH, Y-HsC, C-HL, and Y-MC provided supervision. C-TL, Y-HuC, I-CC, C-HL, and Y-MC were responsible for the validation. C-TL, I-CC, and Y-MC were responsible for visualization and wrote the original draft. C-TL, I-CC, C-HL and Y-MC reviewed and edited the manuscript. Each author contributed important intellectual content during manuscript drafting or revision and agrees to be personally accountable for the individual’s own contributions and to ensure that questions pertaining to the accuracy or integrity of any portion of the work, even one in which the author was not directly involved, are appropriately investigated and resolved, including with documentation in the literature if appropriate. All authors contributed to the article and approved the submitted version.
