# Supplementary figures and images for: The ABCG2 rs2231142 polymorphism and the risk of nephrolithiasis: A case–control study from the Taiwan biobank
Source: Front Endocrinol (Lausanne). 2023 Mar 10;14:1074012. doi: 10.3389/fendo.2023.1074012 (PMC10036833; doi:10.3389/fendo.2023.1074012)

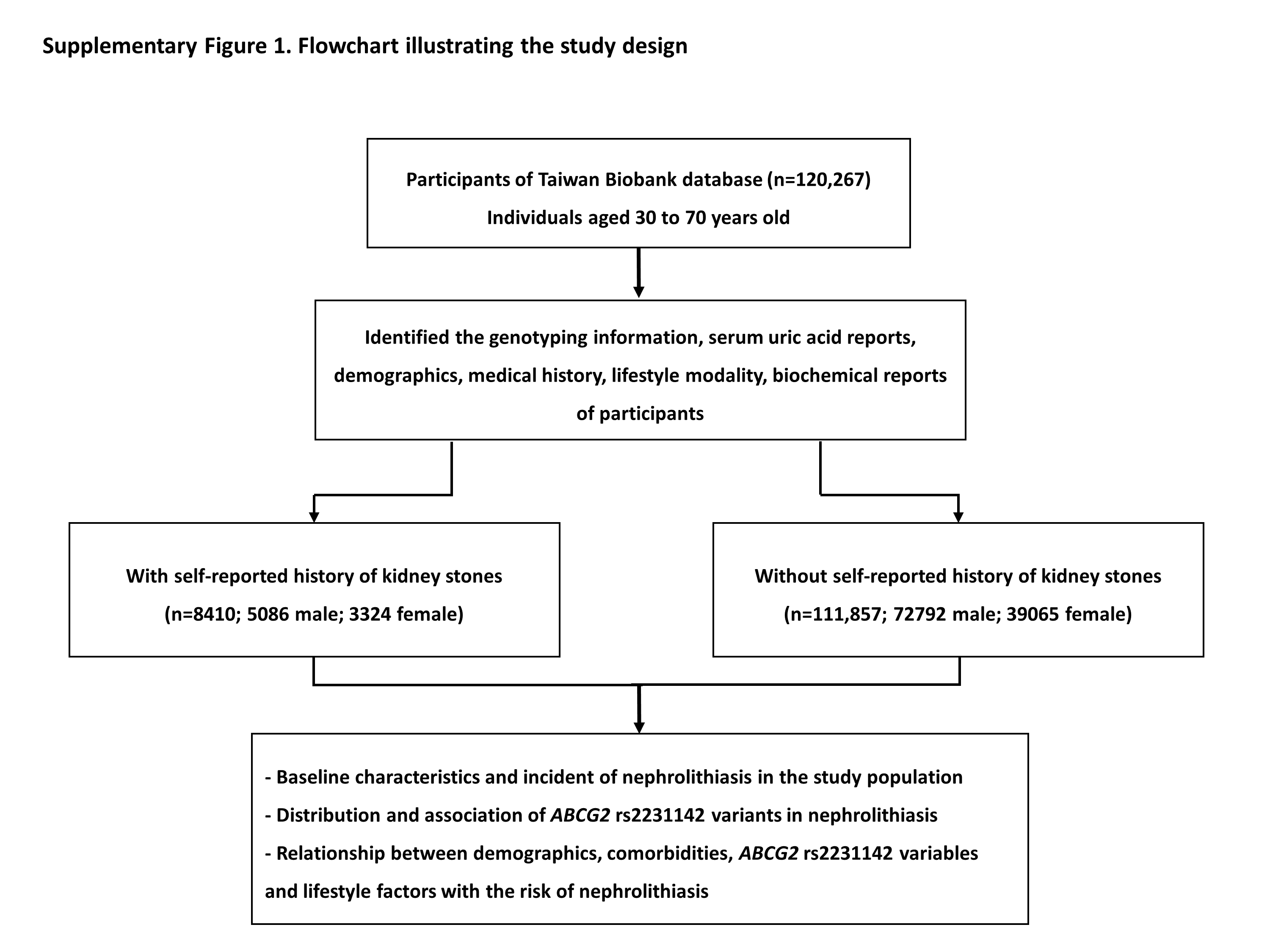

Supplement: Supplementary file 1 [file Image_1.tif]
